# The Batten disease protein CLN3 is important for stress granules dynamics and translational activity

**DOI:** 10.1016/j.jbc.2023.104649

**Published:** 2023-03-24

**Authors:** Emily L. Relton, Nicolas J. Roth, Seda Yasa, Abuzar Kaleem, Guido Hermey, Christopher J. Minnis, Sara E. Mole, Tatyana Shelkovnikova, Stephane Lefrancois, Peter J. McCormick, Nicolas Locker

**Affiliations:** 1 Faculty of Health and Medical Sciences, School of Biosciences and Medicine, University of Surrey, Guildford, United Kingdom; 2 Centre for Endocrinology, William Harvey Research Institute, Barts and the London School of Medicine, Queen Mary, University of London, Charterhouse Square, London, United Kingdom; 3 Centre Armand-Frappier Santé Biotechnologie, Institut national de la recherche scientifique, Laval, Canada; 4 Institute for Molecular and Cellular Cognition, Center for Molecular Neurobiology Hamburg, University Medical Center Hamburg-Eppendorf, Hamburg, Germany; 5 Great Ormond Street, Institute of Child Health and MRC Laboratory for Molecular Cell Biology and Great Ormond Street, Institute of Child Health, University College London, London, United Kingdom; 6 Sheffield Institute for Translational Neuroscience, Department of Neuroscience, University of Sheffield, Sheffield, United Kingdom; 7 Department of Anatomy and Cell Biology, McGill University, Montreal, Canada; 8 Centre d'Excellence en Recherche sur les Maladies Orphelines - Fondation Courtois (CERMO-FC), Université du Québec à Montréal (UQAM), Montréal, Canada

**Keywords:** translation control, stress granules, stress, metabolism, Batten disease, CLN3, NCL

## Abstract

The assembly of membrane-less organelles such as stress granules (SGs) is emerging as central in helping cells rapidly respond and adapt to stress. Following stress sensing, the resulting global translational shutoff leads to the condensation of stalled mRNAs and proteins into SGs. By reorganizing cytoplasmic contents, SGs can modulate RNA translation, biochemical reactions, and signaling cascades to promote survival until the stress is resolved. While mechanisms for SG disassembly are not widely understood, the resolution of SGs is important for maintaining cell viability and protein homeostasis. Mutations that lead to persistent or aberrant SGs are increasingly associated with neuropathology and a hallmark of several neurodegenerative diseases. Mutations in *CLN3* are causative of juvenile neuronal ceroid lipofuscinosis, a rare neurodegenerative disease affecting children also known as Batten disease. *CLN3* encodes a transmembrane lysosomal protein implicated in autophagy, endosomal trafficking, metabolism, and response to oxidative stress. Using a HeLa cell model lacking CLN3, we now show that CLN*3*^KO^ is associated with an altered metabolic profile, reduced global translation, and altered stress signaling. Furthermore, loss of CLN3 function results in perturbations in SG dynamics, resulting in assembly and disassembly defects, and altered expression of the key SG nucleating factor G3BP1. With a growing interest in SG-modulating drugs for the treatment of neurodegenerative diseases, novel insights into the molecular basis of CLN3 Batten disease may reveal avenues for disease-modifying treatments for this debilitating childhood disease.

Batten disease, also known as neuronal ceroid lipofuscinosis (NCL), is a group of fatal inherited neurodegenerative diseases affecting all age groups but predominantly diagnosed in children. Mutations in 1 of 13 distinct genes (*CLN1–CLN8, CLN10–CLN14*) are the cause of NCL (1). The most common form of NCL is juvenile CLN3 disease (2). Symptoms include rapid visual loss, cognitive decline, ataxia, and seizures. First symptoms most often appear between the ages of 5 and 8 years, with death occurring in the third decade of life. The most common mutation found in patients is an intragenic 1-kb deletion spanning exons 7 and 8 (3), resulting in transcripts encoding significantly truncated protein or one lacking multiple internal exons (4). Other less frequent mutations that cause later onset of blindness only have also been identified (1, 5, 6).

CLN3 is a conserved highly glycosylated integral membrane protein with its N- and C-terminal ends located in the cytosol. The protein and its orthologues are localized to various intracellular compartments including endolysosomes (7) and have proposed roles in intracellular trafficking and autophagy (8, 9, 10, 11). CLN3 can interact with Rab7A (12), a small GTPase that regulates the spatiotemporal recruitment of retromer (13, 14). Retromer is a protein complex that is required for the endosome-to-TGN retrieval of the lysosomal sorting receptors, mannose 6-phosphate receptor and sortilin (15, 16). CLN3 can interact with retromer and the sorting receptors and may function as a scaffold protein to ensure efficient interactions (17). In cells lacking CLN3, retromer does not interact with the sorting receptor, which results in their lysosomal degradation (15). This results in inefficient sorting of soluble lysosomal proteins, leading to defective lysosomes. Autophagy defects found in CLN3-deficient cells could be due to the lack of efficient lysosomal protein sorting, the lack of fusion of autophagosome with lysosomes (18), or a combination of mechanisms.

Owing to their highly specialized nature, neurons have a high energy demand, which is met by mitochondrial production of large quantities of ATP by oxidative phosphorylation. Therefore, neurons are especially vulnerable to defects in homeostatic equilibrium and metabolic stresses. Previous studies have suggested that CLN3 is important for maintaining metabolic homeostasis. The loss of CLN3 is associated with the downregulation of oxidative phosphorylation and glycolytic enzymes, and reduced glycolytic metabolites, suggesting impaired glycolysis (19, 20). Moreover, mammalian models of CLN3 disease display altered expression of mitochondrial enzymes involved in cellular respiration, increased accumulation of reactive oxygen species, and membrane depolarization linked to higher cell death levels (21). Studies in *Drosophila* have also revealed a hypersensitivity to oxidative insult for CLN3 mutants (22). Finally, observations in fibroblasts from patients with CLN3 disease have led to the proposal that reactive oxygen species (ROS) accumulation is a common hallmark of and may be correlated with disease severity (23). In addition to these metabolic defects, CLN3 has also been linked to the resolution of endoplasmic reticulum (ER) stress and its resolution *via* the unfolded protein response. The overexpression of wildtype CLN3 appears to be protective against tunicamycin-induced ER stress, increasing expression of ER chaperone glucose-related protein 78 (GRP78) and reducing expression of apoptotic markers such as C/EBP homologous protein (CHOP). In contrast, depletion of CLN3 produced the opposite effect, suggesting its involvement in resolving ER stress (24).

The reorganization of cellular content into stress granules (SGs) is central for cellular adaptation to many stresses including viral infection, protein misfolding, nutrient depletion, UV, heat and oxidative stress. In response to these stresses, the general inhibition of protein synthesis results in the dissociation of mRNAs from polysomes and their accumulation in ribonucleoprotein (RNP) complexes (25). This increased concentration of cytoplasmic RNPs and their binding by aggregation-prone RNA-binding proteins (RBPs), such as Ras-GTPase activating SH3 domain binding protein 1 (G3BP1), results in the recruitment of multiple resident proteins characterized by low sequence complexity and intrinsically disordered regions. These drive clustering/fusion events supported by multivalent interactions between their protein and RNA components, with G3BP1 acting as a key node for promoting RNA–protein, protein–protein, and RNA–RNA interactions, ultimately resulting in liquid–liquid phase separation (LLPS) and SG formation (26, 27). SGs are highly dynamic, rapidly assembling to sequester the bulk content of cytoplasmic mRNAs and dissolving upon stress resolution to release stored mRNAs for future translation (28, 29).

By sequestering specific proteins following stress, SGs alter the composition and concentration of cytoplasmic proteins and therefore have been proposed to modulate biochemical reactions and signaling cascades in the cytosol, impacting on signaling and metabolism (30, 31, 32). Importantly, many signaling molecules associated with diseases can concentrate in SGs, and several are regulated by SGs, supporting a role for SGs as signaling hubs (33). However, because of their proposed stress-specific nature and composition, the exact role for SGs in different stresses remains poorly understood, and while protective functions have been ascribed to SGs, they are also suggested to support prodeath functions (34). SGs are dynamic and reversible, with their disassembly important to restore translation and maintaining protein homeostasis after stress. Mutations impacting protein turnover, or in SG resident RBPs impeding SG clearance or dysregulating LLPS, lead to persistent or aberrant SGs and are emerging as a determinant of neuropathology, in particular in amyotrophic lateral sclerosis (ALS) and related diseases (35). For example, ALS-causing mutations cluster in low-complexity RG-rich regions of the RBP TDP43 or FUS and alter phase transition properties and dynamics of SGs (35, 36). In addition, nonfavorable conditions within the cellular microenvironment typical for a disease state can impair the cells’ ability to respond to additional stressors resulting in the promotion of apoptosis, and the accumulation of ROS inhibits SGs nucleation (36, 37, 38). Collectively these data provide clear evidence linking the pathophysiology of neurodegenerative diseases with SG biology.

With studies linking CLN3 both with metabolic stress and the resolution of ER stress, we set out to investigate the interplay between CLN3 and the SG pathway using a CLN3 knockout (CLN3^KO^) model. We demonstrate that the loss of CLN3 is associated with a defect in mitochondrial activity in HeLa cells, the rewiring of signaling pathways controlling translation, and a reduction of protein synthesis. In turn, CLN3 is important for the assembly of SGs and their resolution upon stress removal, with the accumulation of persistent SGs occurring in CLN3^KO^ cells. These defects in SG assembly/disassembly dynamics are not associated with impaired stress signaling *via* the integrated stress response or a reduced ability of SG resident proteins to phase separate. However, we observed reduced levels of G3BP1 in CLN3^KO^ cells and patient-derived models that could contribute to these defects. These results shed light for the first time on the importance of CLN3 in SG biogenesis and given the recent interest in SGs as druggable targets may provide novel therapeutic opportunities for a rare juvenile disease with currently no efficient treatments.

## Results

### CLN3^KO^ cells display impaired basal metabolic function and are hypersensitive to additional stress

**Figure 1:**
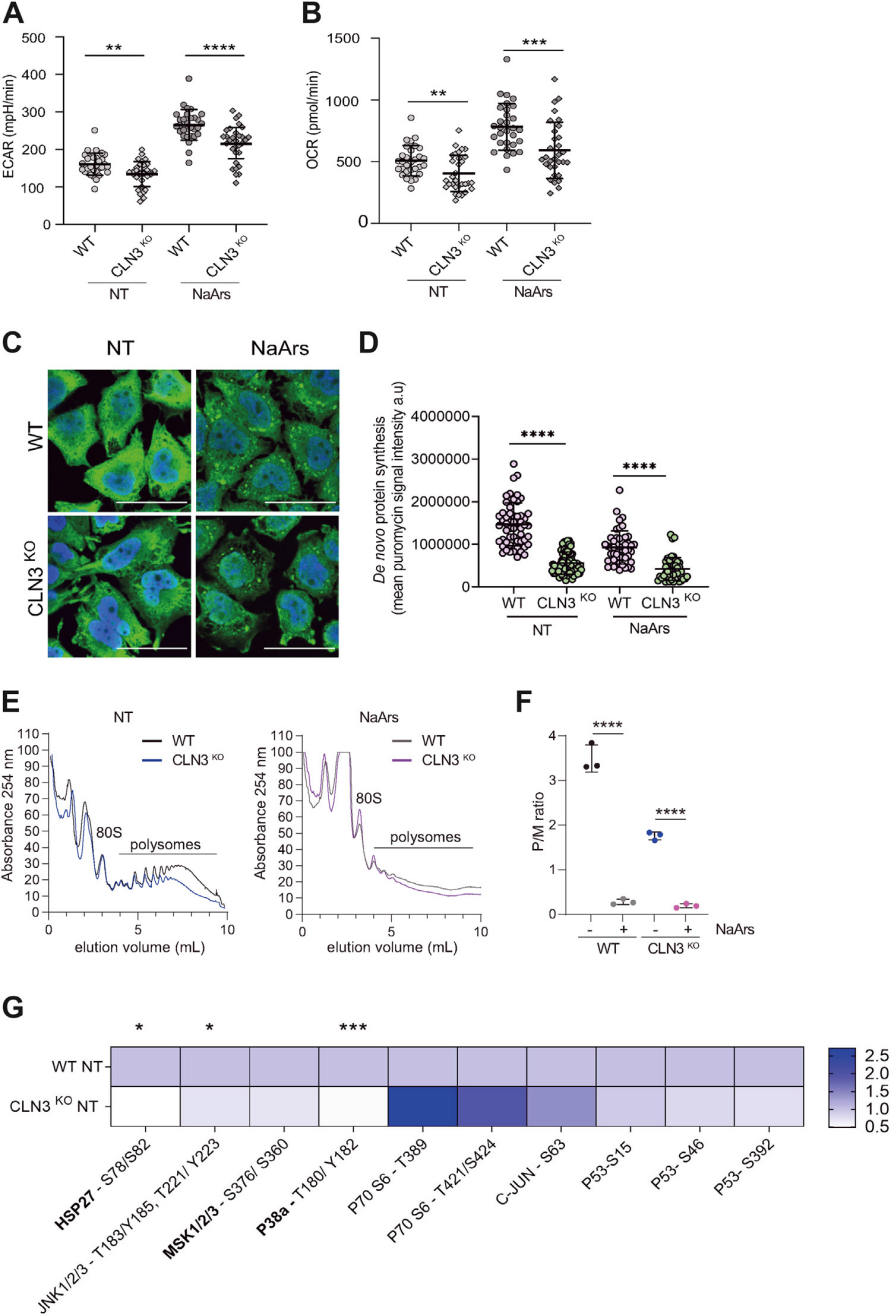
**Loss of CLN3 alters metabolic activity and global protein synthesis.** Metabolic, proteostatic, and signaling defects accompany CLN3 loss in HeLa cells (*A*) efficacy of glycolysis was measured by extracellular acidification rate (ECAR). CLN3^KO^ HeLa cells display reduced glycolytic flux as compared with WT at baseline conditions. The same trend is observed after challenge with NaArs, which increases glycolytic flux irrespective of genotype. The figure shows the mean ± SD, of three separate experiments analyzed by two-way analysis of variance with Dunnett’s multiple comparisons test. *B*, mitochondrial respiration was assessed using oxygen consumption rate (OCR). CLN3^KO^ HeLa cells display reduced OCR under NT (*baseline*) conditions. Challenge with NaArs increases OCR in both genotypes but is significantly lower in CLN3^KO^ HeLa cells. The figure shows the mean ± SD, of three separate experiments analyzed by two-way analysis of variance with Dunnett’s multiple comparisons test: ∗∗*p* < 0.01; ∗∗∗*p* < 0.001. *C* and *D*, efficacy of *de novo* protein synthesis assessed by measuring integrated density values of single cells. Immunofluorescent images of puromycin incorporation (*green*) with nuclei stained with DAPI (*blue*). The scale bar represents 40 μm. The figure shows the mean ± SD, of three separate experiments analyzed by one-way analysis of variance with Sidak’s multiple comparisons test: ∗∗∗∗*p* < 0.001. *D* and *E*, polysome fractions in both WT and CLN3^KO^ HeLa cells in NT condition. CLN3^KO^ HeLa cells exhibit fewer polysomes compared with WT in NT conditions characteristic of reduced rate of translation while NaArs treatment depletes polysome fraction in WT and CLN3^KO^ HeLa cells. Polysomes were prepared as detailed under “Experimental Procedures.” The displayed trace represents absorbance at 254 nm (*vertical axis*) throughout the gradient from *top* (*left*) to *bottom* (*right*). 80 S (monosome) and polysome peaks are labeled. *F*, the areas below the monosome and polysome peaks were determined for several biological replicates (n = 3), and the mean polysome:monosome (P/M) ratio was calculated by measuring the area under the polysomal (P) to monosomal (M) peaks using standard area under the curve methods. The figure shows the mean ± SD, of three separate experiments analyzed by two-way analysis of variance with Dunnett’s multiple comparisons test: ∗∗∗∗*p* < 0.001. *G*, heatmap shows differences in phosphorylation status of a panel of target residues spanning various stress-activated signaling pathways. Decreased phosphorylation of HSP27 (S78/S82), JNK1/2/3 (T183/Y185, T221/Y223), and p38α (T180/Y182). The figure shows the mean ± SD, of three separate experiments analyzed by two-way analysis of variance with Dunnett’s multiple comparisons test: ∗*p* < 0.05; ∗∗∗*p* < 0.001.

As previous studies have shown that the loss of CLN3 is associated with perturbations in metabolic homeostasis (19, 20), we compared the metabolic phenotypes of wildtype (WT) and CLN3^KO^ HeLa cells. The rates of oxidative phosphorylation and glycolysis were determined by measuring the oxygen consumption rate and extracellular acidification rate shown (Fig. 1, *A* and *B*, respectively). CLN3^KO^ cells displayed significantly lower oxygen consumption rate and extracellular acidification rate, suggesting that loss of CLN3 lowers the rates of both glycolysis and mitochondrial respiration by oxidative phosphorylation (*p*-values 0.0019 and <0.0001, respectively). Moreover, after the addition of the oxidative stressor sodium arsenite (NaArs), the difference in the rates of metabolism in the CLN3^KO^ compared with WT HeLa cells was more profound (*p*-values 0.0043 and 0.0007, respectively). These data suggest that CLN3^KO^ cells have an impaired ability to handle additional stressors and start with a lower metabolic turnover in the absence of stressors, which is indicative of impaired mitochondrial function.

### CLN3^KO^ displays hallmarks of impaired protein synthesis

An increasing number of studies support a link between mitochondrial function and proteostasis failure, both defining characteristics of neurodegeneration (39). Given the observed differences in mitochondrial function, we hypothesized that potential alterations in bioenergetics would be deleterious to bulk protein synthesis, a vastly energy-expensive process. To probe the impact of loss of CLN3 on translational control, we assessed global translational efficiency using single cell analysis by measuring the incorporation of puromycin, a tRNA structural homologue that specifically labels actively translating nascent polypeptides and causes their release from ribosomes (40). Anti-puromycin antibodies are then used to detect puromycylated native peptide chains by confocal microscopy (Fig. 1*C*). Quantification of puromycin signal intensity revealed a significant reduction in *de novo* protein synthesis in CLN3^KO^ HeLa cells compared with WT cells under baseline conditions (*p*-values <0.0001 and <0.0001, respectively; Fig. 1*D*). To confirm these results and obtain a deeper understanding of the translational control associated with CLN3^KO^, we used polysome profiling to separate actively translating ribosomes in cytoplasmic extracts. Cell lysate from WT or CLN3^KO^ HeLa cells were prepared and subjected to 15 to 50% sucrose density centrifugation. A typical polysome profile pattern was obtained as shown in Figure 1*E*. As expected, stimulation with sodium arsenite resulted in a total shutdown of translation and disappearance of polysomes (Fig. 1*E*). Furthermore, in contrast to WT cells, CLN3^KO^ cells exhibited a translational defect as shown by a decrease in the amount of polysomes in basal conditions. This was further quantified by monitoring the ratio of polysomes to monosomes. Quantification of the area under the polysome and monosome peak showed that the polysome:monosome (P/M) ratio, reflecting the amount of ongoing translation and mRNAs associated with translating ribosomes, was significantly reduced in CLN3^KO^ when compared with WT cells (*p*-values <0.0001 and <0.0001, respectively; Fig. 1*F*).

### CLN3^KO^ is associated with impaired stress-response signaling

Stress-activated signaling pathways sense perturbations in cellular stoichiometry and drive adaptation to maintain homeostasis. Breakdown in this complex signaling network between organelles is thought to contribute toward the common multifactorial pathologies associated with different neurodegenerative disease (41). We probed the phosphorylation status of effector proteins in CLN3^KO^ HeLa cells across several major stress-activated signaling pathways using a commercially available phospho-mitogen-activated protein kinase array allowing for multiplex membrane-based immunoassays to determine the response to the loss of CLN3 under basal conditions. Our results show differences in the phosphorylation of several stress-activated kinases in CLN3^KO^ HeLa cells (Fig. 1*G*). These include significant decreases in phosphorylation at residues S78/S82 on HSP27, T183/Y185/T221/Y223 on JNK 1/2/3, and T180/Y182 on p38α (*p*-values 0.017, 0.047, and 0.0004, respectively), as well as nonsignificant yet trending increased phosphorylation at residues T389/T421/S424 on P70 S6 kinase. Together, these data suggest that loss of CLN3 may hamper the stress signaling networks involved in sensing and responding to external and internal stressors.

### Stress granule assembly is perturbed by loss of CLN3

**Figure 2:**
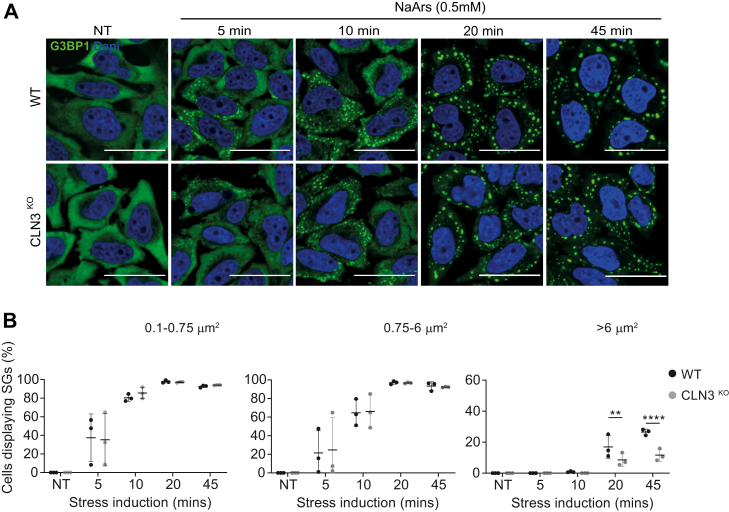
**CLN3**^**KO**^**HeLa cells display defect in stress granule (SG) induction pathway.***A*, representative immunofluorescence images show time course of SG induction after NaArs treatment. Cells were stained with the SG marker G3BP1 (*green*) and the nuclei marker DAPI (*blue*). The scale bar represents 40 μm. *B*, quantification of small (0.1–0.75 μm^2^), medium (0.75–6 μm^2^), and large (>6 μm^2^) puncta shown as % cells displaying SGs. The figure shows the mean ± SD, of three separate experiments, with >100 cells analyzed for each condition, analyzed by one-way analysis of variance with Sidak’s multiple comparisons test: ∗∗*p* < 0.01; ∗∗∗∗*p* < 0.001.

The link between SGs and the pathogenesis of neurodegeneration is well established (35); however, the involvement of SGs in CLN3 disease has not been explored. SGs are important in determining cell fate in response to stressful stimuli, preventing apoptosis by rewiring the stress-activated signaling network (33, 38). Because we observed differences in stress signaling following loss of CLN3, we investigated the ability of CLN3^KO^ HeLa cells to mount an SG response following an acute oxidative insult. NaArs was used to induce SGs over a time course ranging from 5 to 45 min. Cells were then fixed and stained for G3BP1 to detect SG assembly (Fig. 2*A*). First, we confirmed that G3BP1 colocalized with an additional SG marker, eIF3B, suggesting the proper assembly of NaArs-induced canonical SGs in our model cells (Fig. S1). The percentage of cells displaying SGs was quantified using G3BP1 foci as an SG marker, and SGs were categorized as small (0.1–0.75 μm^2^), medium (0.75-6 μm^2^), or large (>6 μm^2^) (Fig. 2*B*). In both cell types, small and medium SGs assembled from 5 min after NaArs treatment and increased with length of NaArs exposure reaching a peak at 20 min of NaArs treatment. Large granules were observed from 20 min, likely due to fusion of smaller granules. However, we observed 50% fewer large SGs at 20 min and 45 min in CLN3^KO^ when compared with WT HeLa cells (*p*-values 0.0028 and <0.0001, respectively), suggesting an impairment in the maturation of SGs in the later stages of SG assembly.

### Loss of CLN3, but not CLN5, is associated with aberrant stress granules disassembly dynamics

**Figure 3:**
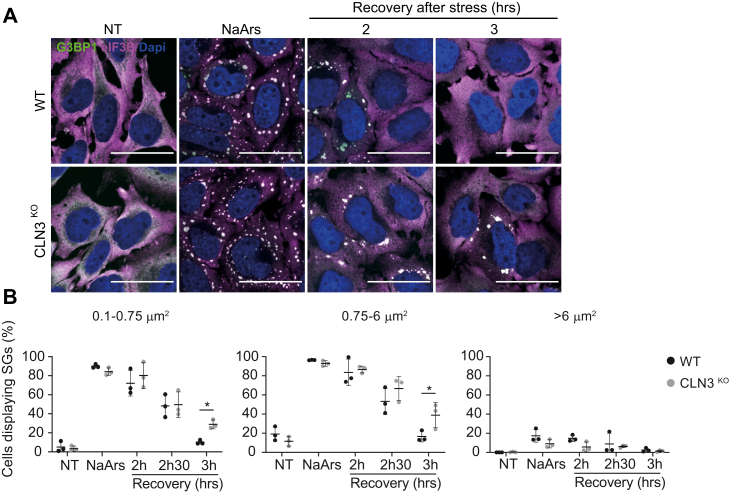
**CLN3**^**KO**^**HeLa cells display defect in clearance of NaArs-induced stress granules (SGs).***A*, representative immunofluorescence images show time course of SG clearance after NaArs challenge. The scale bar represents 40 μm. Cells were stained for SG markers G3BP1 (*green*) and eIF3B (*magenta*). Nuclei were stained with DAPI (*blue*). *B*, quantification of small (0.1–0.75 μm^2^), medium (0.75–6 μm^2^), and large (>6 μm^2^) puncta using ImageJ plugin Aggrecount shown as % cells displaying SGs. The figure shows the mean ± SD, of six separate experiments, with >100 cells analyzed for each condition, analyzed by one-way analysis of variance with Sidak’s multiple comparisons test: ∗*p* < 0.05.

Abnormal turnover of SGs, in particular the persistence of SGs after stress is resolved, is now a well-established hallmark of neurodegenerative disease (35). To assess the impact of loss of CLN3 on SG clearance, cells were stressed with 0.5 mM NaArs for 45 min to induce SGs. The stressor was subsequently washed out, and cells were incubated with fresh medium for a maximum of 3 h. SGs were detected by immunofluorescence using G3BP1 and eIF3b as markers (Fig. 3*A*) and categorized as small, medium, or large as previously described. The frequency of cells containing SGs was then quantified in an automated and nonbiased manner using Aggrecount (37). As expected, NaArs treatment robustly induced the assembly of small and medium SGs, which decreased in frequency in a temporally dependent manner after the removal of the stressor (Fig. 3*B*). By 3 h post stress, the frequency of small and medium SGs had returned to nontreated (NT) levels in WT cells; however, CLN3^KO^ cells displayed altered recovery dynamics. Thirty-five percent of CLN3^KO^ cells had persistent small SGs, and 24% of CLN3^KO^ cells contained persistent medium-sized SGs after 3 h of recovery (*p*-values 0.0363 and 0.0476, respectively). These data suggest that the clearance of NaArs-induced SGs is less efficient in CLN3^KO^ and that CLN3 may be important for recovery from and adaptation to oxidative stress.

Autophagy pathways have been linked to SG clearance (42). Furthermore, it has been previously shown that CLN3 is required for endosome-associated small GTPase RAB7A functions, including its interaction with PLEKHM1, an interaction required for endosome–lysosome and autophagosome–lysosome fusion events, as well as the efficient recycling of lysosomal receptors sortilin and CI-MPR (17) Therefore, we investigated whether impaired SG clearance observed in CLN3^KO^ cells was due to the loss of this CLN3-Rab7A interaction; however, we found SG clearance is unaffected by Rab7A^KO^ HeLa cells (Fig. S2). This suggests that the increased persistence of SGs in CLN3^KO^ is not due to CLN3-dependent Rab7A functions associated with lysosomal function.

**Figure 4:**
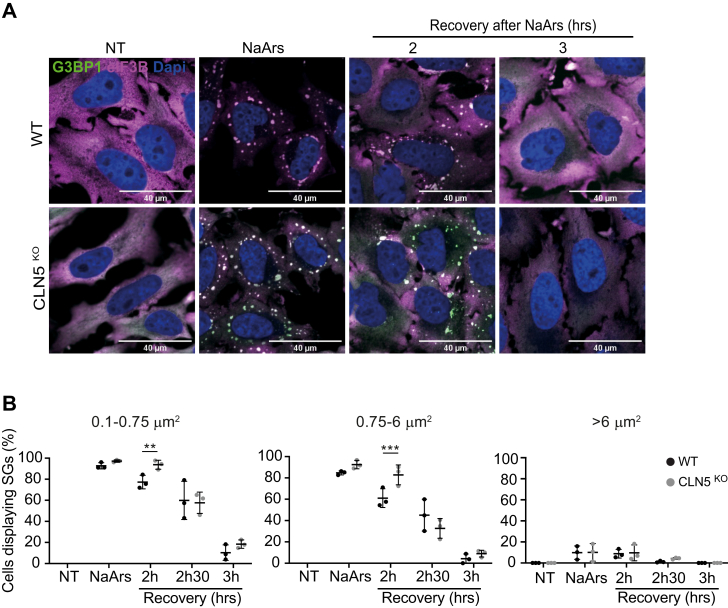
**Impaired stress granule (SG) clearance is a CLN3-specific phenotype.***A*, rate of SG recovery was assessed in an alternative Batten disease model, CLN5^KO^ HeLa cells. Representative immunofluorescence images show time course of SG clearance after NaArs challenge. The scale bar represents 40 μm. Cells were stained for G3BP1 (*green*) and eIF3B (*magenta*). *B*, quantification of small (0.1–0.75 μm^2^), medium (0.75–6 μm^2^), and large (>6 μm^2^) puncta using ImageJ plugin Aggrecount shown as % cells displaying SGs. Results shown as mean ± SD, n = 3. The figure shows the mean ± SD, of three separate experiments, with >100 cells analyzed for each condition, analyzed by one-way analysis of variance with Sidak’s multiple comparisons test: ∗∗*p* < 0.01; ∗∗∗*p* < 0.001.

Batten disease is linked to the dysfunction of several CLN proteins involved in intracellular trafficking (17, 43). To assess if the delay in SG dissolution is CLN3 specific, we investigated SG dynamics in a HeLa KO model of CLN5 disease. NaArs induced small and medium SGs in 97% and 92% of CLN5^KO^ HeLa cells, respectively. After washout of the stressor and a 3-h recovery period, the frequency of all sizes of SGs was comparable between WT and CLN5^KO^ HeLa cells, albeit with a slower start to disassembly observed at 2 h when compared with WT (*p*-values 0.007 and 0.0007, respectively), suggesting that loss of CLN5 does not impact the clearance of SGs and impaired recovery (Fig. 4*A* and *B*).

### Loss of CLN3 does not alter the disassembly of eIF2α-independent SG and also impacts on other biocondensates assembly

**Figure 5:**
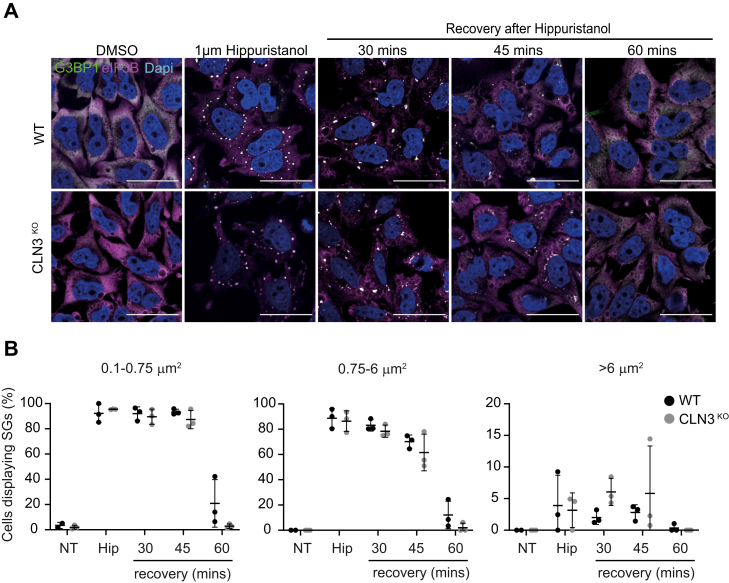
**The dynamics of eIF2α-independent stress granules (SGs) is not affected in CLN3**^**KO**^**HeLa cells.***A*, representative immunofluorescence images show time course of SG clearance after Hippuristanol challenge. The scale bar represents 40 μm. Cells were stained for SG markers G3BP1 (*green*) and eIF3B (*magenta*). Nuclei were stained with DAPI (*blue*). *B*, quantification of small (0.1–0.75 μm^2^), medium (0.75–6 μm^2^), and large (>6 μm^2^) puncta using ImageJ plugin Aggrecount shown as % cells displaying SGs. Results show mean ± SD, n = 6. The figure shows the mean ± SD, of six separate experiments, with >100 cells analyzed for each condition, analyzed by one-way analysis of variance with Sidak’s multiple comparisons test.

In addition to arresting translation *via* eIF2α-dependent pathways, SGs can also assemble following eIF2α-independent inhibition of translation, for example, by blocking eIF4A or mTOR activity (17, 43). Given that SGs induced by eIF2α-dependent or -independent pathways have been proposed to have different functions, we investigated whether the loss of CLN3 also affected the clearance of SGs formed following stimulation with the eIF4A inhibitor Hippuristanol (17, 43). Cells were stressed with 1 μM Hippuristanol for 60 min to induce SGs. The stressor was subsequently washed out, and cells were incubated with fresh medium for 1 h. SGs were detected by immunofluorescence using G3BP1 and eIF3b as markers (Fig. 5*A*) and categorized as small, medium, or large. As expected, Hippuristanol treatment resulted in the assembly of SGs, which disassembled over time following removal of the stressor, reaching the same levels as in NT cells by 60 min. We did not observe any difference between WT and CLN3^KO^ cells (Fig. 5*B*). Therefore, in contrast to NaArs-induced SGs, CLN3 does not seem to be important for recovery from eIF2α-independent stress signaling.

**Figure 6:**
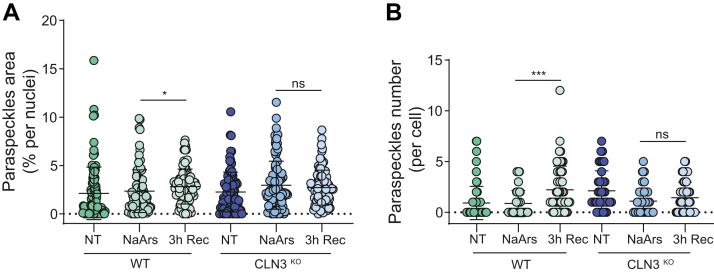
**Paraspeckles dynamics is affected in CLN3**^**KO**^**HeLa cells**. WT and CLN3^KO^ HeLa cells were stained for the paraspeckles marker lcRNA NEAT-1 using RNA FISH after NaArs challenge and quantitated during recovery from stress. Quantification of paraspeckles area per % of nuclei area (*A*), and paraspeckles number per cell (*B*), was performed using ImageJ plugin Aggrecount. The figure shows the mean ± SD, of three separate experiments, with >100 cells analyzed for each condition, analyzed by two-way analysis of variance with Dunnett’s multiple comparisons test: ∗*p* < 0.05; ∗∗∗*p* < 0.001.

Paraspeckles are nuclear RNP granules formed in the interchromatin space, close to splicing speckles and scaffolded (44). The longer isoform of the long noncoding RNA NEAT1, NEAT1_2, is the structural scaffold of paraspeckles. We recently described that paraspeckles and SGs are part of a coordinated biphasic wave of condensate assembly during recovery from stress (45). SGs regulate paraspeckles by sequestering some of their protein components during the acute phase of stress, with paraspeckles hyperassembly being further stimulated once SGs disassemble following stress recovery (45, 46). Therefore, we posited that the impaired disassembly of SGs in CLN3^KO^ cells could also result in altered paraspeckles dynamics. NaArs was used to induce SGs for 45 min, followed by removal of the stressor, and cells were left to recover for 3 h. Cells were then fixed and labeled with NEAT1 RNA-FISH probes to label paraspeckles. The number and area of paraspeckles were then quantified (Fig. 6). In WT cells, as expected, stimulation with NaArs resulted in an increase in size (Fig .6*A*) and numbers (Fig. 6*B*) of paraspeckles by the end of the stress recovery period (*p*-values 0.039 and 0.0001, respectively). In contrast, in CLN3^KO^ cells we did not detect hyperassembly of paraspeckles following removal of the stressor and recovery, with no changes in the number or area of paraspeckles detected (Fig. 6). Therefore, loss of CLN3 impacts on the coordinated wave of biocondensates assembly associated with eIF2α signaling.

### Delayed recovery of SGs is conserved in patient-derived fibroblasts

**Figure 7:**
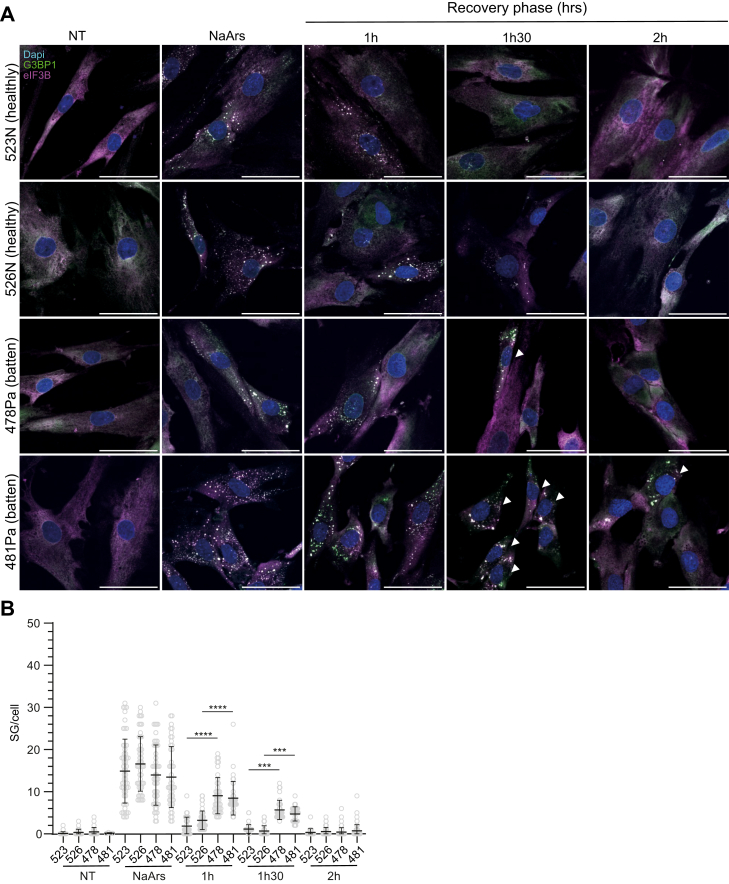
**The impaired clearance of NaArs-induced stress granules (SGs) is conserved in patient-derived fibroblasts.***A*, representative immunofluorescence images show time course of SG clearance after NaArs challenge in two healthy controls and two CLN3 patients-derived fibroblasts (lines 523N and 526N, 478Pa and 481Pa, respectively). The scale bar represents 50 μm. Cells were stained for SG markers G3BP1 (*green*) and eIF3B (*magenta*). Nuclei were stained with DAPI (*blue*). Representative images are shown with white arrows pointing at persistent SGs during the recovery phase. *B*, quantification of small (0.1–0.75 μm^2^), medium (0.75–6 μm^2^), and large (>6 μm^2^) puncta using ImageJ plugin Aggrecount shown as % cells displaying SGs. Results show mean ± SD, n = 6. The figure shows the mean ± SD, of three separate experiments, with >100 cells analyzed for each condition, analyzed by one-way analysis of variance with Sidak’s multiple comparisons test: ∗∗∗*p* < 0.001; ∗∗∗∗*p* < 0.0001.

To further validate these findings in a more relevant cell type, we examined the response of cultured human fibroblasts, from unrelated healthy donors (523N and 526N) or patients with CLN3 disease caused by homozygous 1-kb deletion of CLN3 (478Pa and 481Pa), to stress induction by following SG dynamic in response to NaArs stimulation as described above. Both healthy or patient fibroblasts responded to NaArs stress by assembling SGs as noted by the accumulation of G3BP1 and eIF3b-positive foci (Fig. 7*A*). However, while most of these foci disassembled after 1h30 post stressor removal in healthy donor cells, patients fibroblasts displayed persistent SGs at 1h30 and 2 h post recovery, with a clear reduction in the number of SGs per cell at 1 h30 for both patient lines (*p*-values <0.0001 and 0.0001, and 0.0001 and 0.001, respectively; Fig. 7*B*). Therefore, the altered SG dynamics observed in CLN3^KO^ HeLa cells also occurs in patient-derived models of CLN3 disease, supporting a more general mechanism linking CLN3 and delayed SG recovery.

### Delayed recovery of SGs in CLN3^KO^ cells is independent from eIF2α signaling or chaperones associated with SG disassembly

**Figure 8:**
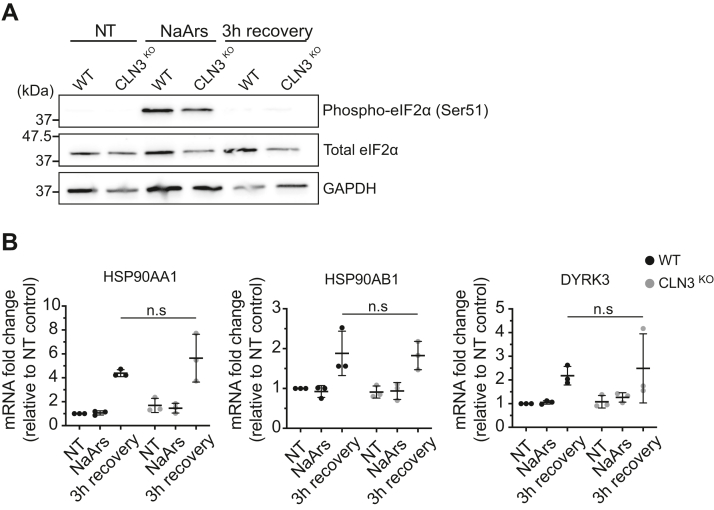
**Stress granule (SG) disassembly defect is not linked to impaired eIF2α signaling or SG chaperones activity.***A*, immunoblotting analysis of eIF2α phosphorylation (Ser51) shows no difference in CLN3^KO^ HeLa cells compared with WT in NT, NaArs treated, and 3 h post NaArs conditions. Molecular weights are indicated to the left (kDa). *B*, RT-qPCR analysis of SG chaperones HSP90AA1, HSP90AB1, and DYRK3 mRNA expression in response to NaArs challenge and 3 h post stress. The figure shows the mean ± SD, of three separate experiments, normalized to β-tubulin mRNA and shown relative to WT NT expression level, analyzed by two-way analysis of variance with Dunnett’s multiple comparisons test: n.s, not significant.

Following transient stress, the dissolution of SGs and translation reentry follows the dephosphorylation of eIF2α (47). Chronic activation of the integrated stress response *via* phosphorylated-eIF2α (p-eIF2α) is associated with SG persistence, toxicity, and neuronal death (48). To assess whether the delayed SG clearance in CLN3^KO^ cells is linked to a defect in eIF2α signaling, p-eIF2α (S51) levels were measured in response to NaArs and at 3 h following stress removal by Western blot analysis. As expected, p-eIF2α (S51) levels increased significantly in WT HeLa cells upon NaArs induction and returned to baseline levels at 3 h following NaArs removal (Fig. 8*A*). NaArs stimulation in CLN3^KO^ HeLa cells resulted in a similar level of p-eIF2α activation, which also returned to baseline levels by the end of the recovery period. These results suggest eIF2α is appropriately dephosphorylated at 3 h post stress in CLN3^KO^ HeLa cells. Therefore, it is unlikely that the increased persistence of SGs observed in CLN3-deleted cells is linked to a defect in eIF2α signaling.

Upon stress, SGs sequester mTORC1 to modulate cell metabolism and growth to subsequently promote stress adaptation. Upon stress relief, HSP90, a molecular chaperone implicated in human disease, stabilizes DYRK3, which drives SG dissolution and the release of mTORC1, enabling restoration of translation and other mTORC1-dependent pathways (49, 50, 51). Aberrant signaling through this axis is associated with persistent SGs and ALS. To probe the impact of CLN3^KO^ on this pathway, we measured the mRNA expression of the stress-inducible and constitutively expressed HSP90 subunits HSP90AA1 and HSP90AB, respectively, as well as the HSP90 substrate DYRK3 in NT, stressed, and 3 h poststress conditions (Fig. 8*B*). As expected, we observed that in WT cells the expression of HSP90AA1 and HSP90B, and DYRK3, wase increased at the end of the recovery period from NaArs stimulation, corresponding to the kinetics of SG disassembly (Fig. 8*B*). A similar pattern was observed in CLN3^KO^ cells, with no significant difference in the chaperone induction between WT and CLN3^KO^ cells. Overall, this suggests that impaired recovery from stress and SG disassembly is not driven by a defect in HSP90 chaperone activity.

### Loss of CLN3 is associated with reduced expression of the SG-scaffolding protein G3BP1 but not its ability to undergo SG-like phase transition

**Figure 9:**
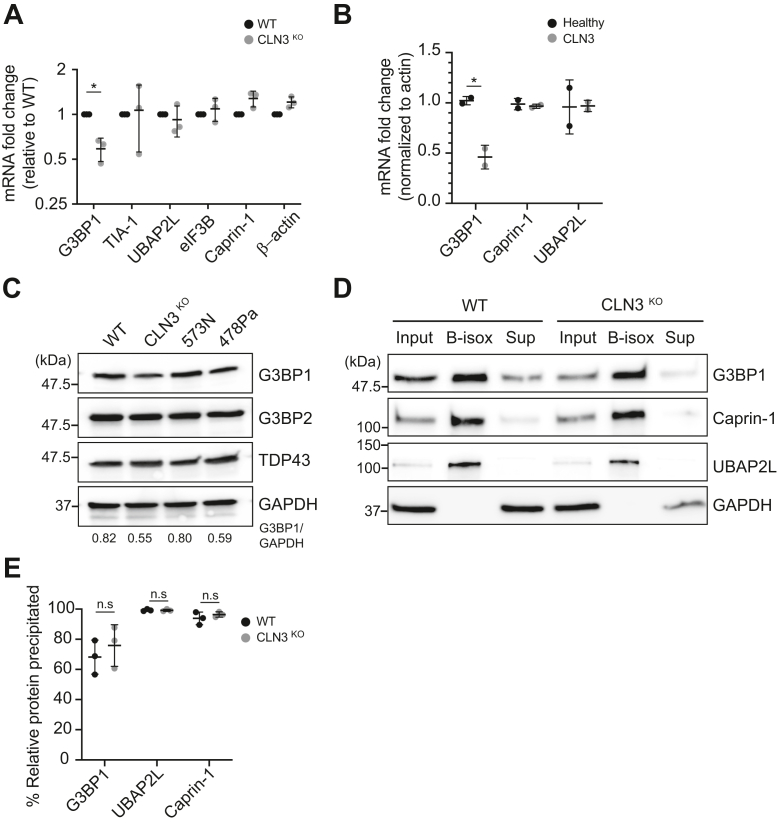
**Reduced G3BP1 levels in loss of G3BP1 models.***A*, RT-qPCR analysis in CLN3^KO^ HeLa cells of stress granule (SG) resident proteins G3BP1, Caprin-1, UBAP2L, TIA-1, and eIF3B as well as β-actin mRNA as control. The figure shows the mean ± SD, of three separate experiments, normalized to β-tubulin mRNA and shown relative to WT NT expression level, analyzed by two-way analysis of variance with Dunnett’s multiple comparisons test: ∗*p* < 0.05. *B*, RT-qPCR analysis of SG resident proteins G3BP1, Caprin-1, and UBAP2L mRNAs in healthy controls or patient-derived fibroblasts (lines 523N and 526N, 478Pa and 481Pa, respectively). The figure shows the mean ± SD, of three separate experiments, normalized to β-tubulin mRNA and shown relative to WT NT expression level, analyzed by two-way analysis of variance with Dunnett’s multiple comparisons test: ∗*p* < 0.05. *C*, Western blot analysis of G3BP1, G3BP2, TDP-43, and GAPDH in WT and CLN3^KO^ HeLa cells, and healthy controls or patient-derived fibroblasts (lines 523N and 478Pa, respectively). The relative levels of G3BP1 in input fraction are shown, normalized to levels in WT cells. Representative picture from three independent experiments, with molecular weights indicated to the left (kDa). *D*, Western blot analysis of distribution of key SG nodes proteins in WT and CLN3^KO^ HeLa cells. For each genotype samples were loaded as input, b-isox pellet, and b-isox supernatant from *left to right*. Representative picture from three independent experiments, with molecular weights indicated to the *left* (kDa). *E*, Graphs show relative precipitation of SG nodes in b-isox pellet compared with input. The figure shows the mean ± SD, of three separate experiments, analyzed by two-way analysis of variance with Dunnett’s multiple comparisons test: n.s, not significant.

Appropriate expression and availability of key SG resident RBPs is a central determinant of SG size and function, with SG size governing functional interactions with other membrane-less organelles (52). Given that CLN3^KO^ cells display significantly fewer large SGs (>6 μm^2^) after NaArs treatment, we investigated whether this was due to altered expression of key SG “node” proteins by measuring the expression of SG proteins G3BP1, TIA-1, UBAP2L, eIF3B, and CAPRIN-1 in untreated conditions (Fig. 9*A*). While the mRNA expression level of most markers is unchanged between WT and CLN3^KO^ HeLa cells, a 2-fold reduction in the expression of G3BP1 is observed in CLN3^KO^ when compared with WT HeLa cells (*p*-value 0.0328). Similarly, fibroblasts derived from patients with CLN3 disease displayed reduced levels of G3BP1 mRNA when compared with healthy controls (*p*-value 0.0126; Fig. 9*B*). Using immunoblotting analysis of WT and CLN3^KO^ cells, healthy control (523N) and patient derived (478Pa), we also observed reduced G3BP1 protein level, but not of its homologue G3BP2, associated with loss of CLN3 (Fig. 9*C*). Reduced G3BP1 mRNA is a feature of ALS with TDP-43 pathology, whereby nuclear depletion of the RBP TDP-43 impairs the stability of G3BP1 transcripts (53). However, we did not observe changes in TDP43. It has been proposed that the assembly of SG is driven by the phase separation of G3BP1 once the percolation threshold has been reached (54). Furthermore, the ability of G3BP1 to undergo phase separation and drive SG assembly in cells under stress is dependent upon its dynamic posttranslational modifications (PTMs), such as the removal of methyl groups on arginine residues and the phosphorylation status of serine 99 (55, 56). These modifications prevent unnecessary and untimely formation of G3BP1 aggregates by neutralizing the physical ability of G3BP1 to homo- and hetero-oligomerize with other SG-associated proteins and nontranslating RNAs. Considering LLPS occurs in a concentration-dependent manner, we asked how the reduced G3BP1 expression observed in CLN3^KO^ cells would affects its propensity for phase separation *in vitro* using biotin isoxazole (b-isox), a surrogate for SG formation (57). B-isox addition, in conjunction with an EDTA–EGTA lysis buffer releasing transcripts from polysomes, results *in vitro* in selective condensation of the low-complexity aggregation-prone proteins *via* phase separation (58). The b-isox-induced aggregates can then be fractionated from the soluble fraction by centrifugation, respectively, defined as pellet and supernatant, and their protein contents analyzed by immunoblotting (Fig. 9*D*). Importantly, this confirmed that total G3BP1 protein levels are reduced in CLN3^KO^ cells compared with WT. Quantification of immunoblotting analysis showed no difference in the levels of G3BP1 recovered in the b-isox precipitated fractions (pellets) compared with the inputs in the extracts from WT or CLN3^KO^ cells (Fig. 9*E*). Moreover, canonical SG markers Caprin1 and UBAP2L were also precipitated with G3BP1, suggesting both the precipitation of low-complexity proteins and the interaction of G3BP1 with nontranslating mRNPs. Overall, these data suggest the reduced level of G3BP1 associated with loss of CLN3 does not impact the ability of other SG proteins to phase separate and condense into SGs.

## Discussion

CLN3 disease displays multifactorial pathologies similar to those seen in age-associated neurodegenerative conditions, including metabolic and calcium signaling abnormalities (59), elevated reactive oxygen species (21), defective intracellular trafficking (17, 43, 60), and autophagy (18). We sought to investigate how loss of CLN3 modulated the SG pathway. We found that cells lacking CLN3 (1) have altered cell metabolism and impaired protein translation, (2) demonstrate delayed SG formation, and (3) display significant delays in SG resolution that are also observed conserved in cells derived from patients with classical juvenile CLN3 disease.

We first confirmed that the CLN3^KO^ HeLa cell model used in this study displayed features of homeostatic imbalance, by assessing rates of glycolysis and oxidative phosphorylation. These data support that loss of CLN3 lowers the basal rates of both glycolysis and oxidative phosphorylation and impairs the ability of cells to handle additional metabolic stress. Thus, our model recapitulates some of the pathological features associated with CLN3 Batten disease. Furthermore, we detected perturbations in stress signaling pathways that are critical for adaptation to adverse cellular environments. We observed reduced activation of the heat shock protein 27 (HSP27), JNK1/2/3, and p38α. HSP27 plays a role in reducing ROS damage, inhibiting various cell death pathways and promoting protein folding is implicated in neurodegenerative diseases (61). Similarly, mitogen-activated protein kinase signaling pathways, specifically JNK and p38α pathways, are activated by environmental and genotoxic stressors and orchestrate multiple cellular defense mechanisms (62). JNK signaling is implicated in neuronal differentiation, motility, metabolism, and apoptosis, and abnormal signaling is linked to neuronal pathology including ALS and Alzheimer’s disease (63). In addition, previous studies using *Drosophila* models have demonstrated a genetic interaction between CLN3 and the JNK signaling pathway (22) and revealed a role for the orthologue Btn1 in response to stress using fission yeast *Schizosaccharomyces pombe* (64). Together, our CLN3^KO^ HeLa cells display altered homeostasis at basal levels, sensitivity to additional metabolic stress, and altered stress signaling, which supports a role for CLN3 in the cellular stress response.

In response to stressful stimuli, SGs sequester nontranslating mRNA, RBPs, and signaling molecules to regulate a network of cellular pathways to regain homeostasis. Given we have observed metabolic, signaling, and proteostasis defects and an increased sensitivity to additional stressors, we probed the ability of CLN3^KO^ cells to mount an SG response to exogenous stress caused by sodium arsenite. Surprisingly, this set of experiments revealed that significantly fewer large SGs are present at the later stages of stress induction in CLN3^KO^ compared with WT HeLa cells. SG assembly occurs in a step-wise manner when translation initiation is stalled. First, nontranslating mRNP complexes oligomerize into stable core structures. Second, cores act as scaffolds for the recruitment of further mRNP complexes that undergo many fleeting and promiscuous interactions with one another resulting in the formation of the outer dynamic shell and phase separation from surrounding cytoplasm. Third, during the latter stages of assembly, small SGs undergo multiple fusion events to form larger, mature SGs that contain multiple cores (65). The reduction in large SGs observed during the later stages of assembly may be due to impairments in the fusion of small SGs that form early in the stress response. Small assemblies can be directly or indirectly transported along microtubules during cell transport. Studies have shown that stress-induced RNA granules can be tethered to motile lysosomes for long distance travel (66). One possibility is that defective lysosome function and localization (12) in CLN3 disease impairs lysosome motility, which directly effects the ability of RNA granule to “hitchhike” during cell transport, in turn preventing fusion events that lead to granule maturation. Future experiments should aim to further investigate this interaction between SG dynamics and lysosomes.

To further probe the molecular mechanism that could explain the defect in SG assembly, we assessed the levels of several SG markers in CLN3 ^KO^ cells. This revealed that the key SG nucleating factor G3BP1 was downregulated by 2-fold at the mRNA level in CLN3^KO^ cells, while other transcripts encoding proteins important within the SG core were unaffected. G3BP1 encodes a multifunctional RBP that interacts with multiple cell pathways including stress response and RNA metabolism, suggesting its importance for maintaining homeostasis (67). In SG formation, G3BP1 is thought to be a critical regulator of LLPS due to its high valency for RNA binding and therefore reduced G3BP1 amounts could contribute to defects in the SG dynamics associated with loss of CLN3 (27).

SG dynamics is influenced by a tightly regulated network of protein quality control (PQC) pathways that maintain protein homeostasis by promoting the timely disassembly of SGs after stress. Recent work on SG disassembly has shown it is a highly context-specific process, with stressor type and duration influencing the mechanism of clearance (68, 69). Failure of PQC pathways is associated with aging and disease and leads to persistent SGs, a putative cause of neurodegeneration (70). Our data showed an increased persistence of small and medium SGs after stress release associated with CLN3^KO^ cells, which could not simply be explained by prolonged eIF2α activation. Interestingly, these defects in SG clearance are specific to SG induced by eIF2α-dependent pathways, which may reflect the stress-specific nature of SGs, proposed to be compositionally and structurally heterogeneous depending on the stress (70). In addition, during recovery from stress, SGs control the hyperassembly of the nuclear biocondensate paraspeckles, which are important for RNA splicing. In consequence, we observed reduced hyperassembly of paraspeckles in CLN3^KO^ cells reflecting the broad impact of CLN3 on cytoplasmic and nuclear biocondensates.

SG integrity is also maintained by a network of molecular chaperones that prevent protein misfolding within the granule and aid the recruitment of other essential SG factors. Heat shock protein 90 (HSP90) is a major chaperone implicated in SG dissolution and stress adaptation. By modulating the activity of dual-specificity tyrosine-phosphorylation-regulated kinase 3 (DYRK3), HSP90 couples SG dissolution with the release of mTORC1 and reinitiation of metabolism. Reduced activity and expression of DYRK3 is seen in ALS and is causative of SG clearance defects and decreased viability (51). Although we observed an increase in expression of HSP90 subunits during the 3-h recovery phase, there was no difference in the change of expression between WT and CLN3^KO^ cells, suggesting that, in our model, HSP90 modulation of DYRK3 is intact and any impairment in SG clearance is not due to inactivation of DYRK3. Recent work has shed light on the role of PTMs in condensate assembly (27, 71). In particular, for the key SG node G3BP1, phosphorylation at residues S149 and S232 flanking its acidic intrinsically disordered region has been proposed to increase the threshold for phase separation likely keeping G3BP1 in an SG-incompetent conformation (26, 27). In addition, the G3BP1 RGG domain is the target of differential arginine methylation by the protein arginine methyltransferases PRMT1 and PRMT5, with methylation at residues R435, R447, and R460 strongly inhibiting SG formation, while loss of PRMT1 and PRMT5 activity is associated with a small SG phenotype (55). Together, PTMs of G3BP1 represent a way of fine-tuning SG assembly, and future studies should investigate whether loss or overexpression of CLN3 has any effect on these.

The equilibrium of cellular protein levels (termed proteostasis) is maintained by a network of interactions between numerous biochemical pathways. Protein synthesis, folding, PTM, and degradation are highly regulated to ensure the functional requirement of cells are met while ensuring PQC and degradation pathways are not overwhelmed (72, 73). Exposure to stress can perturb the delicate balance of interactions that maintain proteostasis, which may subsequently lead to pathological compensatory changes. Equally, reduced efficiency of degradation pathways by disease-associated mutations or aging can result in the accumulation of toxic protein aggregates (74). Measuring translation at single cell level, combined with polysomes profiling, we observed a significantly reduced rate of translation under basal conditions in CLN3^KO^ cells, a reduced association of mRNAs with highly translating ribosomes, and a reduced polysome:monosome ratio in CLN3^KO^ cells. Overall, this suggests there are fewer mRNA-bound ribosomes in steady-state conditions and impaired translational activity associated with loss of CLN3. Recent work in the fission yeast model has shown a genetic interaction between the CLN3 orthologue, *btn1*, and genes encoding ribosomal proteins suggesting a link between CLN3 function and protein synthesis (75). Work in the same HeLa KO system used in this study has also shown CLN3^KO^ impairs the late stages of autophagy (17, 18). Autophagy is one of the major catabolic routes of protein degradation and essential for cell viability. Impairment in the clearance of proteins disturbs the balance of protein homeostasis, and cells may establish compensatory mechanisms to restore equilibrium. It is plausible that, in CLN3^KO^ cells, global translation is reduced to lessen the burden on a deficient autophagy pathway. Data from the literature further support this, as previous work has shown a direct and functional interaction between CLN3 and Rab7a, a small GTPase implicated in the function of late endosomes (17). Late endosomal Rab7a has been shown to bear mRNA that is translated by associated ribosomes and translational machinery. These complexes of endosomes and RNP granules dock onto mitochondria and translate mRNA essential for mitochondrial vitality (76). Mutations causing CLN3 disease disturb the distribution of late endosomal and lysosomal compartments resulting in perinuclear clustering and impaired motility (12). We suggest that the physical interaction between late endosome/lysosome and translating RNP granules may be impaired in CLN3^KO^ cells. Furthermore, decreased site-specific translation of mitochondrial proteins, which is dependent on this interaction, might contribute to impaired mitochondrial activity. Together, these data suggest that the requirement for CLN3 in translation is conserved between yeast and mammalian cells. It remains unanswered what mRNAs are being translationally repressed, whether it is a global phenomenon or specific to certain populations of transcripts. Future work expanding on the polysome fractionation performed in this study may shed light on this question and unravel the deregulated pathways that contribute toward CLN3 disease pathogenesis.

This study has shown for the first time a potential interaction between CLN3 pathology and SG assembly, disassembly, and the regulation of steady-state translation. Given the role of SGs in the reorganization of cellular contents to maintain homeostasis in steady-state conditions and in response to stressful stimuli, and recent work highlighting the importance of lysosomes in SG shuttling in neurons to avoid neurodegeneration, our study paves the way for further investigation in patient-derived cells to explore the link between lysosomes, SG dynamics, and modulation in CLN3 disease.

## Experimental procedures

### Cell culture

HeLa WT, CLN3, and CLN5 cell lines were maintained in Dulbecco's modified Eagle's medium (DMEM) supplemented with 100 U/ml penicillin, 100 g/ml streptomycin, and 10% fetal bovine serum. at 37 °C in a humidified chamber at 95% air and 5% CO2. CLN3^KO^, CLN5^KO^, and Rab7A^KO^ HeLa cells have been described (17, 43, 77); the same parental line was used to generate these KO lines (17, 43, 77). Fibroblast cells were derived from skin biopsies taken from two unrelated healthy individuals (523N and 526N) and from two unrelated patients (478Pa and 481Pa) homozygous for the 1-kb deletion that causes classic juvenile CLN3 disease. Cells were seeded at a density of 5 × 10^6^/well in 24-well plates, 2 × 10^6^/well in 6-well plates, and 10 × 10^6^/well in 15-cm dishes.

### Stress induction and recovery assays

For SG induction assay, stress was induced with 0.5 mM sodium arsenite (NaArs, Sigma) for 5, 10, 20, or 45 min at 37 °C. For the SG recovery assay, granules were induced with the addition of 0.5 mM NaArs for 45 min. NaArs was then washed out with fresh DMEM for 5 min at 37 °C, and this process was repeated 3 times. Fresh medium was added for the final time and cells were allowed to recover for a maximum of 3 h. Hippuristanol stress was induced in a similar manner using 1 mM Hippuristanol (kind gift from Jerry Pelletier, McGill University) and 1h30 recovery period. Immunofluorescence staining and image acquisition was carried out as indicated below. All images were processed and analyzed using ImageJ software package Fiji (http://fiji.sc/wiki/index.php/Fiji), and plugin Aggrecount (https://aggrecount.github.io) was used to quantify SGs in an automated and nonbiased manner (37). GraphPad prism was used for data presentation, and analysis was performed using multiple *t* tests.

### Ribopuromycyclation assay

Quantification of *de novo* protein synthesis was performed as described in (53). To capture translation efficacy in NT (baseline) and stressed conditions, cells were treated with 0.5 mM NaArs for 45 min. Then, prior to fixation, cells were treated with 10 μg/ml of puromycin (Sigma) for 5 min at 37 °C to label the nascent polypeptide chains before addition of 180 μM of emetine (Sigma) to block the translation elongation with a further incubation of 2 min at room temperature (RT). Cells were rinsed with warm DMEM, then fixed with 200 μl 4% paraformaldehyde (PFA) for 20 min at RT, and washed 3 times in PBS. Puromycin incorporation was quantified using ImageJ software package Fiji. The raw integrated density values were recorded for ∼100 cells per condition. GraphPad prism was used for data presentation, and analysis was performed using Mann Whitney U test.

### Polysomes fractionation

Cells were seeded to 10-cm^2^ plates. Upon reaching ∼80% confluency, cells were treated with 100 μg/ml of cycloheximide (CHX, Sigma) for 3 min at 37 °C to stall translating ribosomes onto the transcripts before being placed on ice, washed twice with cold PBS(−) (Invitrogen) containing 100 μg/ml CHX, then scraped into 15-ml falcon tubes, pelleted by centrifugation, snap frozen in liquid nitrogen, and stored at −80 °C. To separate polysomes, samples were layered onto a 10 to 50% sucrose gradient in lysis buffer and centrifuged in an SW40Ti rotor (Beckman Coulter) at 38,000 rpm for 2 h. Gradients were fractionated into 1-ml fractions using a FoxyR1 collection system (Teledyne ISCO), and UV absorbance was monitored at 254 nm. To induce runoff of polysomes, CHX was omitted from the lysis and gradient buffers and replaced with 10 mm EDTA.

### Biotin isoxazole precipitation

The protocol was performed as described in (52). Briefly, cells were seeded to 10-cm^2^ plates at 2 × 10ˆ6 cells per plate 24 h prior to experiment. Cells were placed on ice and washed twice with cold PBS (from 10× solution–Lonza). While in PBS, cells were gently disassociated using a cell scraper and transferred to a 15-ml falcon tube and centrifuged at 2000 RPM RT for 3 min. The supernatant was removed, and pellet was snap frozen in liquid nitrogen. Cells were lysed on ice in cold EE buffer (Hepes pH 7.4 50 mM, NaCl 200 mM, Igepal 0.1%, EDTA pH 8 1 mM, EGTA pH 8 2.5 mM, Glycerol 10%, DTT 1 μM supplemented with RNAsin [Promega]). Lysates were transferred to 1.5-ml tubes and incubated with agitation for 20 min at 4 °C, then centrifuged at 13,000 RPM 15 min at 4 °C. A volume of 50 μl of the supernatant was kept as “input,” mixed with an equal volume of 2× Loading buffer (Cell Signalling) and boiled at 95 °C for 5 min. The remaining supernatant was supplemented with 100 μM of b-isox (Sigma), and the precipitation reactions were carried out for 90 min at 4 °C with agitation. A volume of 50 μl supernatant was kept as “soluble fraction” and mixed in equal volume with 2x loading buffer and boiled 95 °C for 5 min. The pellet containing precipitated aggregates was rinsed twice in cold EE buffer and spun at 10,000 RPM 10 min at 4 °C, then resuspended in 200 μl x1 loading buffer as “insoluble fraction” and boiled at 95 °C for 5 min. Fractions were further analyzed by Western blot performed as indicated. Quantification was performed by calculating relative precipitation of protein in b-isox pellet compared with input. Data were analyzed in GraphPad Prism, and statistical analysis was performed using unpaired *t* test.

### Phosphokinase arrays

Cells were seeded to 15-cm^2^ plates at 5 × 10ˆ6 cells per plate 48 h prior to experiment. Prior to harvesting, cells were treated with 0.5 mM NaArs for 45 min and harvested straight away by scraping into a 15-ml tube, followed by centrifugation and snap freezing the pellet or washed 3 times in DMEM to remove NaArs and incubated for further 3 h before harvesting. Cell pellets were lysed in 1 ml lysis buffer from Human phosphokinase array kit (R&D systems, ARY003C) supplemented with protease inhibitor cocktail (Roche). Protein concentration was measured with BCA pierce kit (Fisher), and 400 μg protein used for each analysis. Arrays were performed as per manufacturer’s guidelines. Images were acquired using the VILBER imaging system and quantified in FIJI. Data presentation and analysis was performed in GraphPad prism using multiple *t* tests.

### Immunoblotting

Cells were plated at 2 × 10^5^ in 6-well plates. At the indicated times, cells were lysed in 150 μl of 1× Gel Loading Buffer (New England Biolabs), sonicated and boiled 5 min at 95 °C. Cell lysates were separated by SDS-PAGE, and protein was transferred to nitrocellulose or polyvinylidene difluoride membranes. These were then probed with the following primary antibodies: rabbit anti-eIF2a (1:1,000, Cell Signalling), mouse anti-P-eIF2α (1:1,000, Cell Signalling), rabbit anti-G3BP1 (1:2,000, Sigma), rabbit anti-Caprin1 (1:1,000, Bethyl Laboratories), goat anti-eIF3B (1:2,000, Santa Cruz), mouse anti-GAPDH (1:20,000, Invitrogen), followed by incubation with the appropriate peroxidase-labeled secondary antibodies (Dako) and chemiluminescence development using the Clarity Western ECL Substrate (Bio-Rad). The results were acquired using the VILBER imaging system.

### Immunofluorescence

A total of 5 × 10^5^ HeLa cells were plated on glass coverslips in a 24-well plate and fixed with 4% PFA (Sigma) in PBS for 20 min at RT, washed in PBS, and stored at 4 °C. Cells were permeabilized with 0.1% Triton-X100 (Sigma) in PBS for 7 min, then blocked with 0.5% bovine serum albumin (Fisher) in PBS for 1 h. Coverslips were then incubated with 200 μl of primary antibody solution for 1 h at RT and washed 3 times with PBS before incubation with secondary antibody solution containing 0.1 μg/ml DAPI solution (Sigma) for 1 h at RT. After three washes with PBS, coverslips were mounted to microscope slides with 7 μl Mowiol 488 (Sigma). Confocal microscopy was performed on a Ti-Eclipse A1MP Multiphoton Confocal Microscope (Nikon) using the Nikon acquisition software NIS-Elements AR. Primary antibody dilution: Rabbit anti-G3BP1 (1:600, Sigma), Goat anti-eIF3B (1:600, Santa Cruz), mouse anti-puromycin (1:5, http://dshb.biology.uiowa.edu/PMY-2A4), Rabbit anti-UBAP2L (1:600, Santa Cruz). Secondary antibodies were all purchased from Invitrogen: Chicken anti-mouse Alexa 488, donkey anti-goat Alexa 555, and goat anti-rabbit 488.

### RNA fluorescence *in situ* hybridization

HeLa cells were seeded to coverslips in 24-well plates at a density of 5 × 10^4^ per well 24 h before experiment. Briefly, stress was induced using NaArs for 45 min, and cells were allowed to recover for 3 h. Cells were fixed with ice-cold 4% PFA for 15 min, washed once with 1x PBS, then permeabilized with 75% ethanol at 4 °C for up to a week. Ethanol was removed and coverslips were rinsed twice with 2× saline-sodium citrate (SSC) buffer before adding hybridization buffer (20% formamide in 2× SSC) for 5 min at RT. Forty-microliter droplets of hybridization buffer containing probe (Stellaris FISH Probes Human NEAT1 Middle Segment with Quasar 570 Dye) were dispensed onto parafilm and coverslips were positioned on top, then incubated at 37 °C in a dark humidified chamber overnight. On the next day coverslips were transferred back to plate and incubated with hybridization buffer in the dark at 37 °C for 15 min, then washed in 1× PBS for 15 min. Nuclei were stained with DAPI for 5 min before washing with 1× PBS. Coverslips were mounted to microscope slides with a drop of Mowiol-488.

### Reverse transcription quantitative polymerase chain reaction

Prior to experiment cells were seeded at a density of 2 × 10^5^ cells/well in 6-well plates. Cells were washed in cold PBS, then total RNA was isolated using quick-RNA extraction mini prep kit (Zymo Research) according to the manufacturer’s instructions before quantification on Nanodrop. Reverse transcriptions were carried out on 0.5 μg of purified RNA using the Precision nanoScript2 Reverse Transcription kit (Primer Design), and quantitative PCR was performed on 5 μl of the cDNA libraries diluted at 1:5 using the Precision Plus 2× 598 qPCR Mastermix (Primer Design) according to the manufacturer’s instructions and the Quant Studio 7 Flex (Applied Biosystems).

Primer list.

**Table:** 

Gene	Forward (5′-3′)	Reverse (5′-3′)
G3BP1	GCCTGTTGCTGAACCAGAGCCT	TGGACGGGGCTGTGAAGCTG
Eif3b	CGGTGCCTTAGCGTTTGTG	CGGTCCTTGTTGTTCTTCTGC
TIA-1	CGAGATGCCCAAGACTCTATACG	CCTTACCCATTATCTTCCGTCCA
PABP	AGACGGAACTTAAGCGCAAA	CGACCACCCTCCATCATAAC
CAPRIN-1	TCTCGGGGTGATCGACAAGAA	CCCTTTGTTCATTCGTTCCTGG
UBAP2L	ATTCGCCTCACTCTCCACAC	TACCACCACACAACACAGCA
β-Actin	TCACCCACACTGT*G*CCCATCTACG	CAGCGGAACCGCTCATTGCCA ATG
B-Tubulin	CTGAACCACCTTGTCTCAGC	AGCCAGGCATAAAGAAATGG
HSP90AA1	ATGGCAGCAAAGAAACAC	GTATCATCAGCAGTAGGGTCA
HSP90AB1	TGGCAGTCAAGCACTTTTCTGT	GCCCGACGAGGAATAAATAGC
DYRK3	TCCTTCTGAACCACCTCCAC	CCTTCATCTCACCTCCATCC

### Seahorse methods

The measurement of rates of oxidative phosphorylation and glycolysis in wildtype HeLa cells and CLN3 KO HeLa cells was carried out using the Agilent Seahorse XF Cell Energy Phenotype Kit (Agilent #103325-100) using the Agilent Seahorse XFe 96 Analyzer, according to the manufacturer’s instructions. WT or CLN3 KO HeLa cells were plated into the Seahorse XF Cell Culture Microplate at a concentration of 20,000 cells/well in DMEM (Gibco #41966-029) + 10% fetal bovine serum (Sigma #F9665). Seahorse XF Assay medium was supplemented with 1 mM pyruvate, 2 mM glutamine, and 10 mM glucose (Agilent # 103681-100). Oligomycin and FCCP were reconstituted to concentrations of 10 μM and 5 μM, respectively. The metabolic phenotype was measured using the Cell Energy Phenotype assay template. Data were normalized by protein concentration using the Protein Assay kit (Bio-Rad #5000116). GraphPad Prism was used for data presentation, and statistical analysis was performed using the unpaired *t* test.

### Statistical analyses

Statistical analyses were performed by using the GraphPad Prism software. Statistical significances were calculated by performing one- or two-way analysis of variance with ad hoc corrections for multiple comparisons tests with a minimum of three biological replicates for analysis.

## Data availability

All the data are included in the article. All unique reagents generated in this study are available from the corresponding authors with a completed Materials Transfer Agreement.

## Supporting information

This article contains supporting information.

## Conflict of interest

The authors declare that they have no conflicts of interest with the contents of this article.

## Supporting information

Sup Figure 1

Sup Figure 2
