# Supplementary figures and images for: The Batten disease protein CLN3 is important for stress granules dynamics and translational activity
Source: J Biol Chem. 2023 Mar 24;299(5):104649. doi: 10.1016/j.jbc.2023.104649 (PMC10149212; doi:10.1016/j.jbc.2023.104649)

Supplementary figure 1

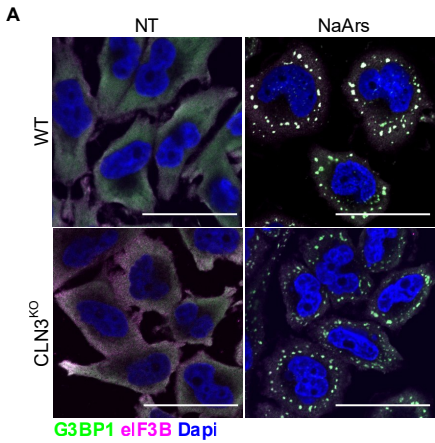

Supplement: Sup Figure 1 [file mmc1.pdf]

Supplementary figure 2

A

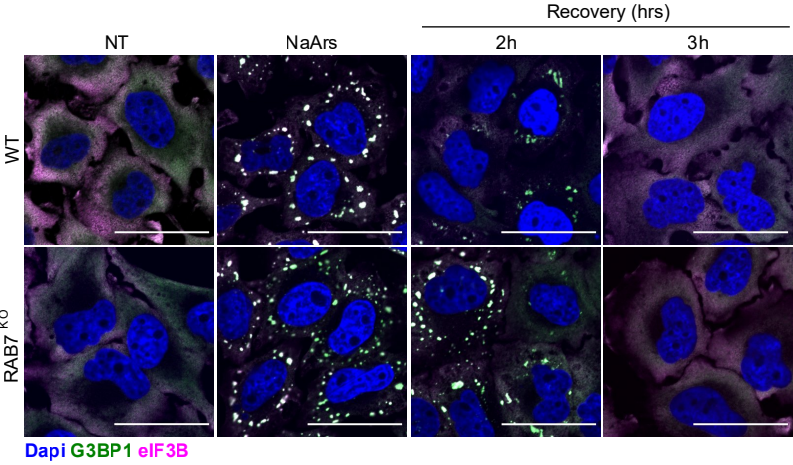

Supplement: Sup Figure 2 [file mmc2.pdf]
